# Isodeoxyelephantopin from *Elephantopus scaber* (*Didancao*) induces cell cycle arrest and caspase-3-mediated apoptosis in breast carcinoma T47D cells and lung carcinoma A549 cells

**DOI:** 10.1186/1749-8546-9-14

**Published:** 2014-04-17

**Authors:** Farha Arakkaveettil Kabeer, Geetha Balakrishnan Sreedevi, Mangalam Sivasankaran Nair, Dhanya Sethumadhavannair Rajalekshmi, Latha Panickaparambil Gopalakrishnan, Remani Prathapan

**Affiliations:** 1 Division of Cancer Research, Regional Cancer Centre, Thiruvananthapuram, Kerala, India; 2 Jawaharlal Nehru Tropical Botanic Garden and Research Institute, Thiruvananthapuram, Kerala, India; 3 CSIR-National Institute for Interdisciplinary Science and Technology, Thiruvananthapuram, Kerala, India

## Abstract

**Background:**

Isodeoxyelephantopin (IDOE) isolated from *Elephantopus scaber* L. (*Didancao*) is used in Chinese medicine for the treatment of some types of cancer. The anti-cancer mechanism of IDOE remains unclear. This study aims to investigate the antiproliferative activity of IDOE on breast carcinoma T47D cells and lung carcinoma A549 cells.

**Methods:**

The growth inhibitory effects of IDOE on breast carcinoma T47D cells, lung carcinoma A549 cells, and normal lymphocytes were evaluated by the MTT assay. Morphological analysis of apoptosis induction was performed by acridine orange/ethidium bromide dual-staining and Hoechst 33342 nuclear staining. The cell cycle profile, caspase-3 expression, and annexin V staining were evaluated by flow cytometry.

**Results:**

IDOE inhibited the growth of A549 and T47D cells in a dose- and time-dependent manner with IC_50_ values of 10.46 and 1.3 μg/mL, respectively. IDOE was not significantly toxic to normal lymphocytes. The cells became detached from the monolayer and rounded up, had fragmented nuclei and condensed chromatin, and the numbers of apoptotic cells increased (*P* = 0.0003). IDOE-induced cell death was associated with activated caspase-3 expression followed by cell cycle arrest at G2/M phase.

**Conclusions:**

IDOE inhibited the proliferation of breast cancer cells and lung carcinoma cells and induced caspase-3-mediated apoptosis and cell cycle arrest in the treated cells.

## Background

*Elephantopus scaber* L. (Asteraceae) is a well-known Chinese medicine that is widely used in the treatment of nephritis, edema, dampness, chest pain, fever and cough of pneumonia, and scabies in Tropical Africa, Eastern Asia, Indian Subcontinent, Southeast Asia, and Australia [1]. Infusion and decoctions of the whole plant are used to stimulate diuresis, reduce fever, and eliminate bladder stones [2]. Extensive phytochemical investigations of the plant isolated different secondary metabolites such as flavonoids, lignans, terpenoids, elephantopin, triterpenes, stigmasterol, epifriedelinol, lupeol, and sterols [3]. Recently, anticancer activity of *E. scaber* has been reported for different types of human tumor cell lines [4].

*E. scaber* has been used in Chinese medicinal with hepatoprotective, anticancer, and antibacterial activities [5-7]. The plant has been reported to contain sesquiterpene lactones, deoxyelephantopin, Isodeoxyelephantopin (IDOE), and scabertopin [8], and the IDOE isolated from the plant exhibited cytotoxicity toward SMMC­7721, HeLa, and Caco­2 cells with IC_50_ values of 18.28, 14.59, and 18.28 μM, respectively, after 48 h of treatment [9]. IDOE was also found to inhibit the growth of KBM-5 human chronic myeloid leukemia cells by altering transcription factor NF-kB expression [10].

However, its modes of actions on lung adenocarcinoma and breast carcinoma cell lines have not yet been elucidated. This study aims to investigate the antiproliferative activity of IDOE on breast carcinoma T47D cells and lung carcinoma A549 cells.

## Methods

### Materials and reagents

DMEM, RPMI 1640 medium, PI, ribonuclease-A, Triton X-100, 3-(4,5-dimethylthiazol-2-yl)-2,5-diphenyltetrazolium bromide (MTT), and Hoechst 33342 were obtained from Sigma Chemical Co. (St. Louis, MO, USA). Dimethyl sulfoxide (DMSO) was purchased from Merck Co. (Darmstadt, Germany). Fetal bovine serum (FBS) was obtained from Gibco BRL (Grand Island, NY, USA). The solvents used for purification and spectroscopic studies (AR grade) and Silica gel were obtained from Merck, Germany. Experimentation procedures described including maintenance of cell lines were reviewed and approved by institutional Ethics Committee (**Regional Cancer Centre**, Trivandrum, Kerala).

### Drugs

**Figure 1:**
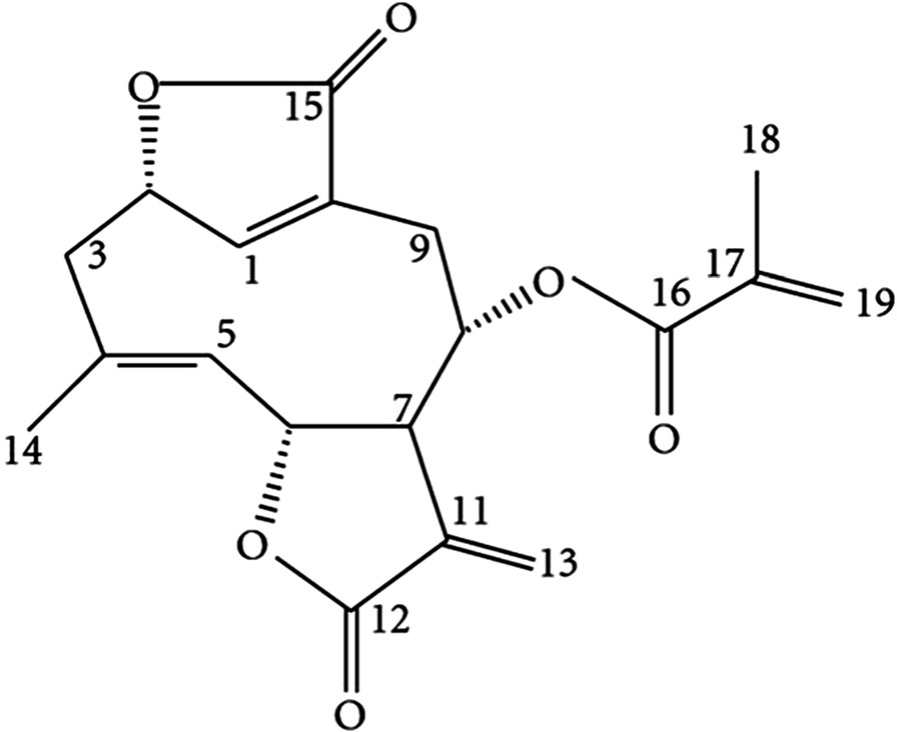
Chemical structure of IDOE.

IDOE (Figure 1) with a purity of 99% was isolated, purified, and identified from *E. scaber* as previously described [11]. Briefly, fresh *E. scaber* whole plants were dried, powdered, and extracted with chloroform for 12 h. The chloroform extract was concentrated, subjected to silica gel column chromatography, and eluted with hexane and a gradient of hexane–ethyl acetate. The fractions eluted with 15% ethyl acetate in hexane were purified by column chromatography and eluted with hexane and a gradient of hexane–ethyl acetate. IDOE was crystallized from the fractions eluted with 10% ethyl acetate in hexane. The structure of IDOE was elucidated by infrared spectrometer (Bruker FT IR, Bruker Optik GMBH, Germany), ^1^H, and ^13^C NMR spectrometry (Bruker AMX 500 MHz NMR, Bruker, Switzerland) and confirmed by the spectral data and melting point of the compound reported in the literature [8]. IDOE was dissolved in DMSO at a concentration of 10 mM and stored at -20°C. Dilutions of IDOE were made in culture medium immediatedly before the experiments. Paclitaxel (Sigma, St. Louis, MO, USA) was used as a positive control.

### Cell culture

Lung adenocarcinoma A549 and breast carcinoma T47D cell lines were obtained from the National Centre for Cell Sciences (India). The cells were cultured in DMEM containing 10% FBS and maintained at 37°C in a 5% CO_2_ environment.

Adult human peripheral blood samples were drawn for isolation of normal human lymphocytes. The blood specimens were diluted 1:1 with phosphate-buffered saline (PBS) (Merck, Germany) and normal lymphocytes were separated by a standard Ficoll-Paque Plus gradient method (GE Healthcare, Pittsburgh, USA). Normal lymphocytes were resuspended in RPMI- 1640 medium with 10% FBS for cytotoxicity assay.

### Cytotoxicity assay

Cell viability was assessed by the MTT assay. The cells were seeded in 96-well plates at 5 × 10^3^ cells/100 μL/well. IDOE concentrations ranging from 0–25 μg/mL for A549 cells and 0–5 μg/mL for T47D cells were added. The plates were incubated at 37°C for 24, 48, and 72 h. MTT (5 mg/mL) was then added to each well and incubated in the dark for 2 h at 37°C. Lysis buffer (100 μL) was added to each well and incubated for 4 h to dissolve the formazan crystals produced. The absorbances of the wells were measured using a microplate reader (Biotek, USA) at a wavelength of 570 nm. The growth inhibition was assessed using the following equation:

$$\begin{array}{ll} \%\;\mathrm{of}\;\text{growth}\;\text{inhibition}= & \left[100-\left(\text{absorbance}\;\mathrm{of}\;\text{treated}\;\text{cells}\right)\right)\\ & \left(/\;\text{absorbance}\;\mathrm{of}\;\text{control}\;\text{cells}\right)\\ & \left(\times \;100\right] \end{array}$$

The respective half-maximal inhibitory concentration (IC_50_) values after 48 h of incubation were determined for each cell line. Normal lymphocytes (2 × 10^4^ cells/100 μL) were seeded in 96-well plates. IDOE was added at concentrations ranging from 0–35 μg/mL and incubated for 72 h. The viability of lymphocytes was measured by the same procedure described above.

### Morphological changes of tumor cells observed by light microscopy

A549 and T47D cells were seeded in 96-well plates (2 × 10^4^ cells/well) and incubated overnight to allow adhesion. The cells were treated with IDOE at concentrations of 10.46 and 1.3 μg/mL for 48 h. Morphological changes were observed by phase-contrast microscopy (Model IX51; Olympus, Japan).

### Acridine orange/ethidium bromide staining

The morphology of apoptotic cells was analyzed under a fluorescence microscope (Model IX51; Olympus, Japan) by staining IDOE-treated cells with acridine orange and ethidium bromide.

### Hoechst 33342 staining

Morphological analyses of apoptosis were performed by staining the cells with Hoechst 33342. The treated A549 and T47D cells were washed twice with PBS and stained with Hoechst 33342 for 1 h at room temperature. The Hoechst-stained nuclei were visualized by a fluorescence microscope (Model IX51; Olympus, Japan).

### Annexin V-FITC/propidium iodide (PI) double-staining assay

Annexin V-FITC/propidium iodide (PI) double-staining was performed with an Annexin V-FITC Kit (BD Bioscience, USA). A549 and T47D cells were treated with IDOE at concentrations of 10.46 and 1.3 μg/mL for 48 h. The cells were trypsinized, rinsed twice with PBS, and resuspended in 1× binding buffer. The cells were labeled with 5 μL of FITC-conjugated annexin V and 5 μL of PI according to the manufacturer's instructions. After incubation in the dark for 15 min at room temperature, 400 μL of binding buffer was added and the samples were immediately analyzed with a flow cytometer (Becton Dickinson, San Jose, CA). The annexin V-FITC^-^/PI^-^ population was regarded as normal, while the annexin V-FITC^+^/PI^-^ and Annexin V-FITC^+^/PI^+^ populations were taken as measurements of early and late apoptotic cells, respectively.

### Detection of caspase 3 expression

Caspase-3 expression was evaluated with a FITC-conjugated Anti-caspase-3 Antibody Detection Kit (BD Bioscience, USA) in accordance with the manufacturer’s instructions. IDOE-treated tumor cells (1 × 10^6^ cells) were collected, washed in PBS, resuspended in 500 μL of BD cytofix solution, and incubated for 20 min on ice. The cells were stained with antibody solution (20 μL of antibody in 100 μL of wash buffer) and incubated for a further 30 min at room temperature. The samples were analyzed by flow cytometry.

### Cell cycle analysis

Following IDOE exposure for 48 h, 1 × 10^6^ cells were collected, washed in PBS, fixed in 70% ethanol, and kept at 4°C overnight. The cell pellet was then washed again with PBS, resuspended in 200 μL of cold PBS, and stained with PI solution [0.01% Triton X-100, 10 μL RNase A (10 mg/mL) and 20 μL PI (1 mg/mL)] for 30 min in ice. The total cellular DNA content was analyzed by flow cytometry (Becton Dickinson, San Jose, CA).

### Statistical analysis

Data were presented as the mean ± standard deviation. The IC_50_ values were determined by the EasyPlot program. Statistical analysis was performed using Graph Pad Prism 5 (GraphPad Prism software Inc., San Diego, USA). Difference between two groups was analyzed by two-tailed Student’s *t* test, and that between three groups was analyzed by one-way ANOVA followed by Tukey’s multiple comparison test. A *P* value of < 0.05 was considered statistically significant.

## Results

### IDOE suppresses the growth of tumor cells without affecting lymphocytes

**Figure 2:**
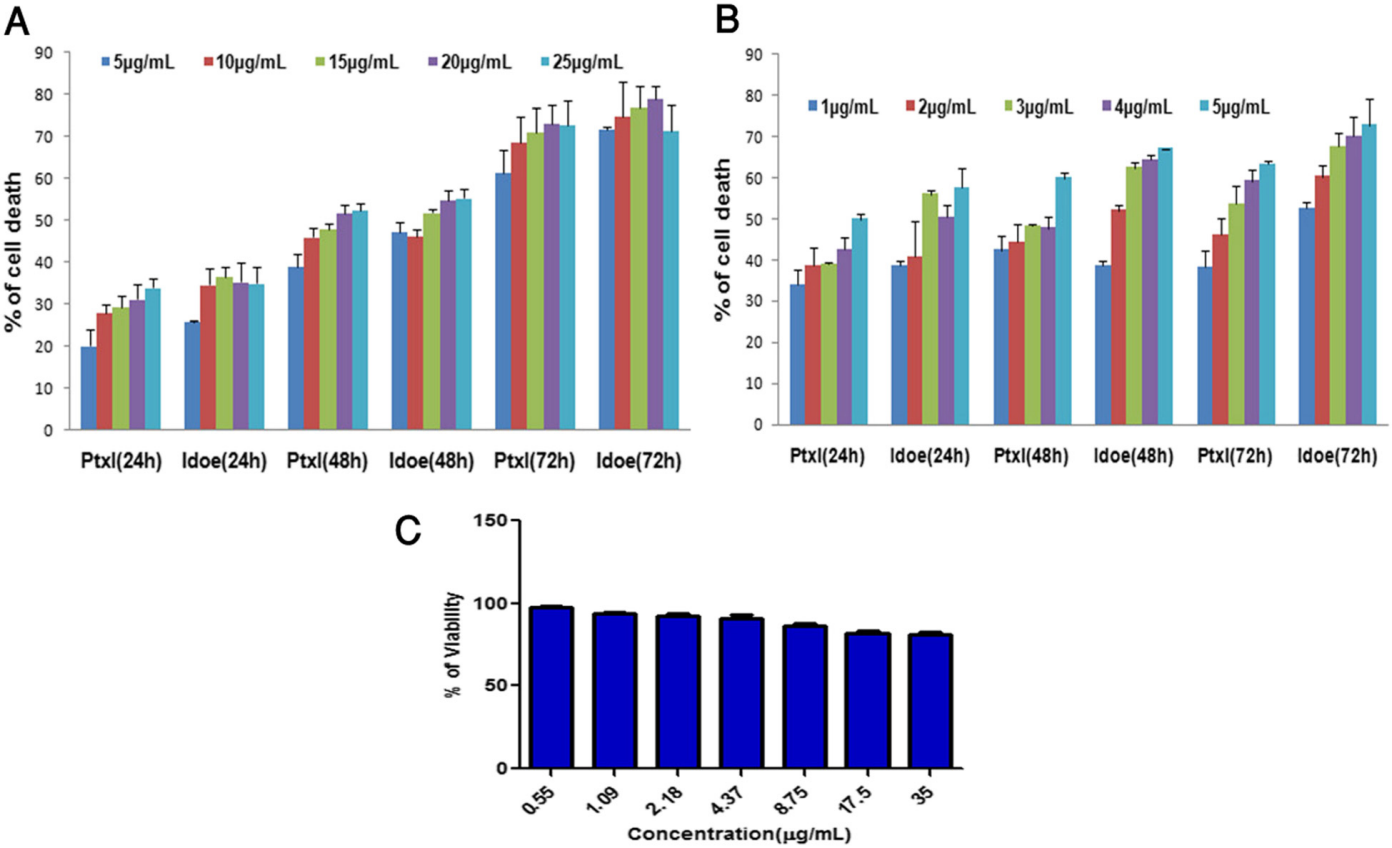
**Effect of IDOE on the cell viability of (A) A549 (B) T47D and (C) Normal Lymphocytes.** Cells were treated with IDOE at various concentrations for 24, 48 and 72 h and cell viability was determined by MTT assay. Paclitaxel (Ptxl) was used as positive control. *** *P* < 0.001 compared to the control.

In comparisons, the cell growth inhibition effect of IDOE was more effective on T47D cells than on A549 cells (Figure 2A and B). The IC_50_ values were 10.46 μg/mL for A549 cells and 1.3 μg/mL for T47D cells. Longer exposure of the cells to IDOE resulted in lower concentrations required to achieve the IC_50_ in both A549 and T47D cells (*P* < 0.001). The determined IC_50_ values were used for the subsequent experiments. IDOE was less toxic toward peripheral blood lymphocytes (Figure 2C).

### Morphological analysis of tumor cells by light microscopy

**Figure 3:**
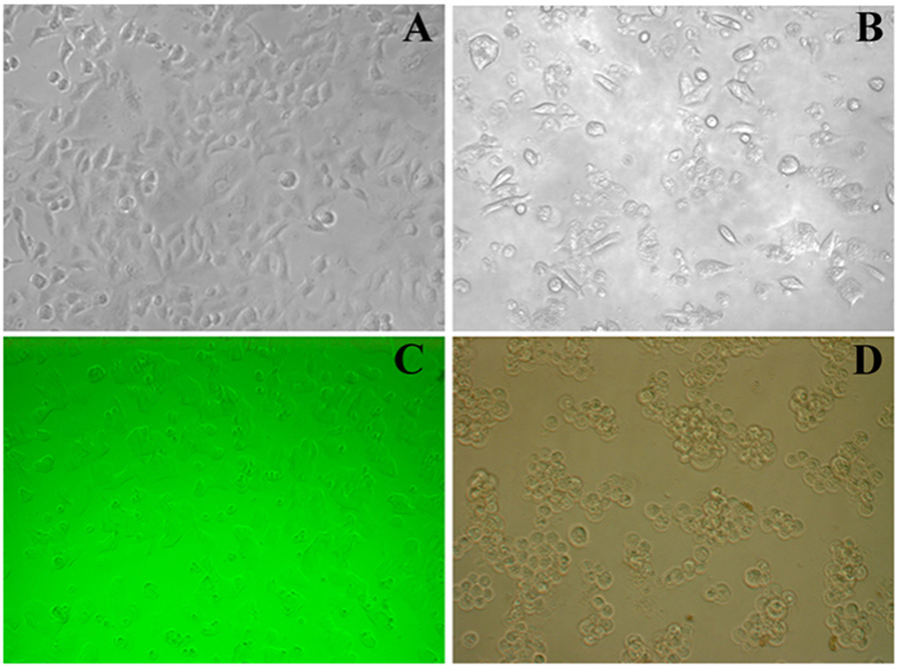
**Analysis of morphological changes of IDOE treated tumor cells (A) Untreated A549 control cells, (B) A549 cells treated with 10.46 μg/mL IDOE, (C) Untreated T47D control cells, and (D) T47D cells treated with 1.3 μg/mL IDOE.** The cells were exposed to indicate concentrations of IDOE and morphological changes were observed after 48 h of treatment. The photographs were taken with an inverted microscope at × 20 magnification.

After 48 h of incubation with IDOE, many of the cells showed cytoplasmic shrinkage and loss of normal nuclear architecture, became detached from the flask, and were floating in the medium (Figure 3).

### Fluorescence microscopic analysis of apoptotic cells

**Figure 4:**
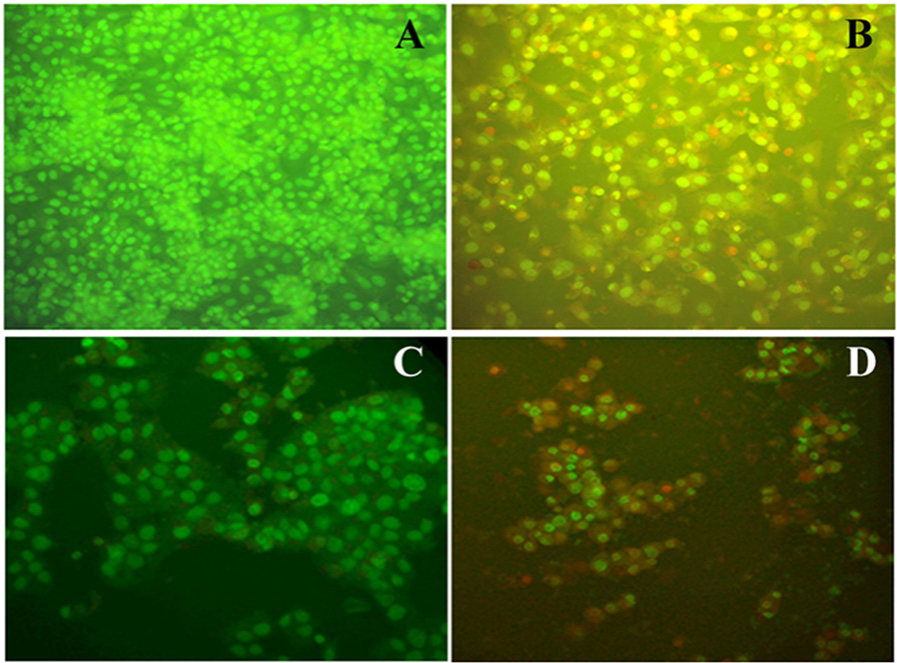
**Acridine orange/ethidium bromide double staining to analyze the apoptotic morphology of IDOE treated tumor cells. (A)** Untreated A549 control cells, **(B)** A549 cells treated with 10.46 μg/mL IDOE, **(C)** Untreated T47D control cells, and **(D)** T47D cells treated with 1.3 μg/mL IDOE. Viable cells (bright green nuclei); early apoptotic cells (bright green condensed nuclei); late apoptotic cells (red nuclei with condensed or fragmented chromatin) under fluorescent microscopy.

As shown in Figure 4, acridine orange/ethidium bromide staining revealed the characteristic features of apoptosis. The cytoplasm and nucleus of normal cells appeared green under fluorescence microscopy, suggesting that IDOE was able to trigger cell death through apoptosis.

**Figure 5:**
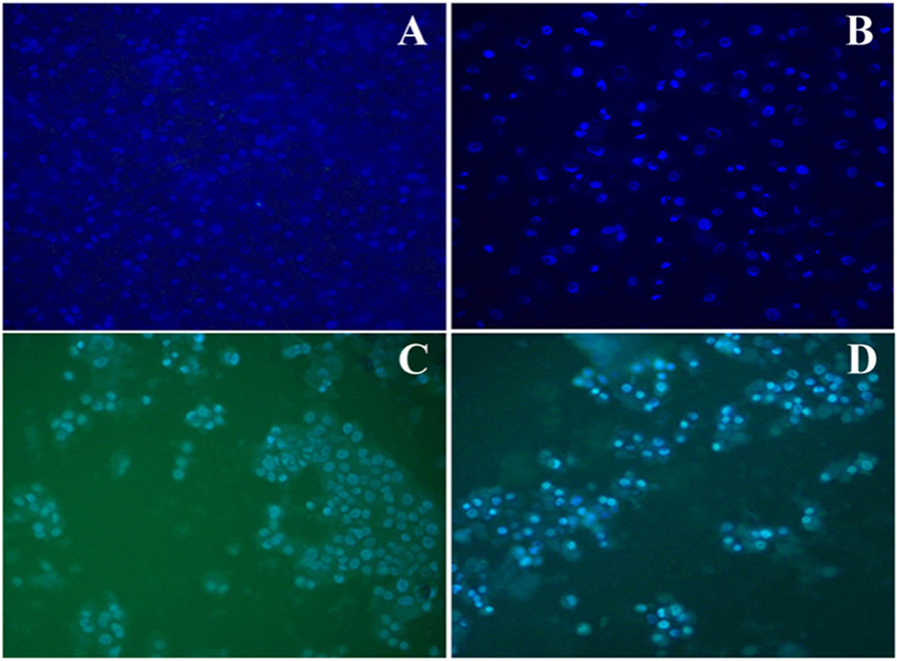
**IDOE-induced apoptosis in tumor cells. (A)** Untreated A549 control cells, **(B)** A549 cells treated with 10.46 μg/mL IDOE, **(C)** Untreated T47D control cells, and **(D)** T47D cells treated with 1.3 μg/mL IDOE. The cells were treated with IDOE for 48 h, stained with Hoechst 33342 (10 μM) and examined by fluorescence microcopy at a magnification of × 20.

The IDOE-treated cells stained with Hoechst 33342 (10 μM) revealed condensed chromatin, fragmented nuclei, and nuclear shrinkage (Figure 5). The number of apoptotic cells increased after treatment with IDOE for 48 h (*P* = 0.0003).

### Annexin V staining of IDOE-treated cells

**Figure 6:**
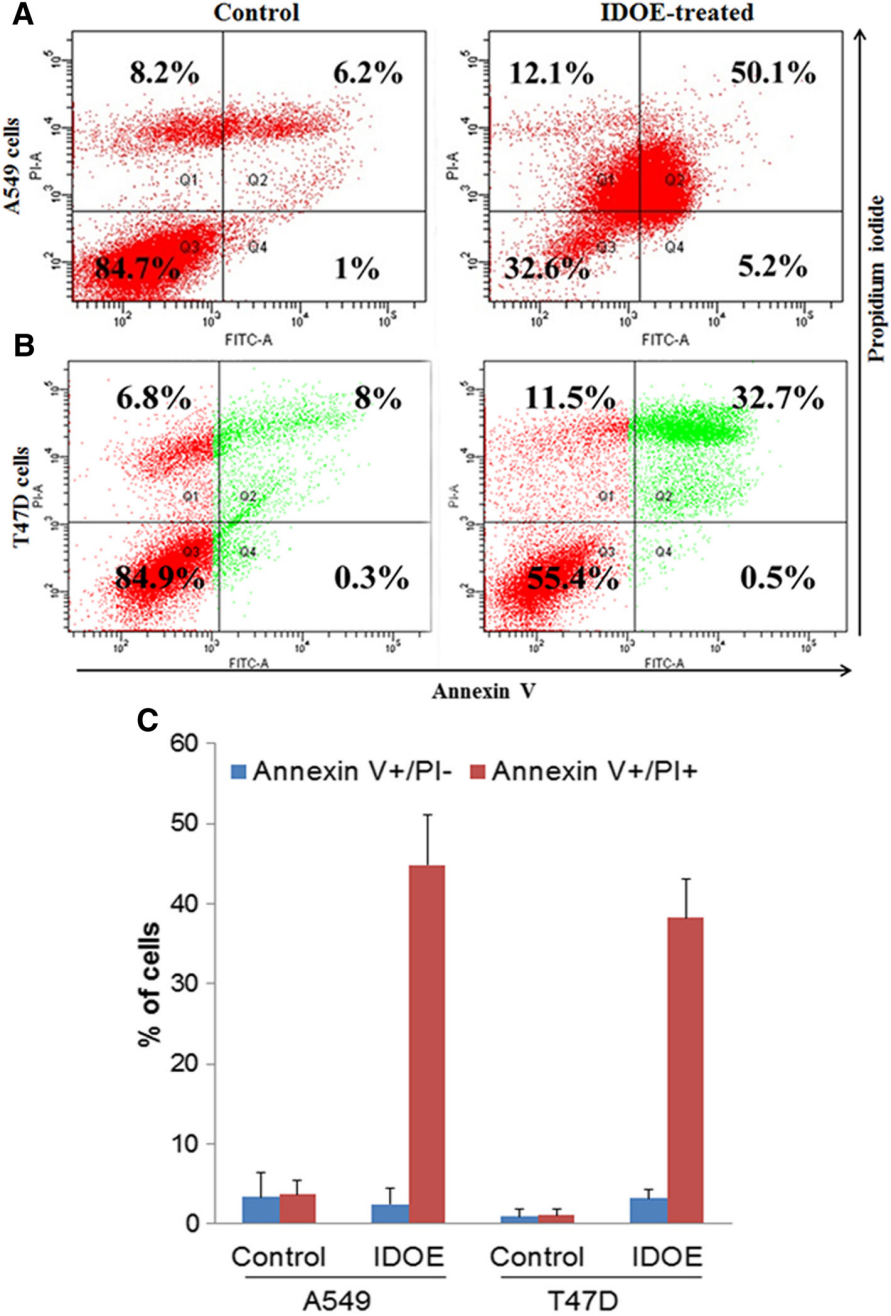
**Flow cytometric analysis of Annexin V and propidium iodide staining in cells treated by IDOE (A) A549 cells (B) T47D cells.** The viable cell populations are in the lower left quadrant (Annexin V-/PI-), the cells at the early apoptosis are in the lower right quadrant (Annexin V+/PI-), and the ones at the late apoptosis are in the upper right quadrant (Annexin V+/PI+). **(C)** Data expressed as mean ± SD from three independent experiments. *P* < 0.05 *vs* control.

In annexin V staining, control A549 cells showed 1% early apoptotic cells and 6.2% late apoptotic cells, whereas IDOE-treated A549 cells showed 5.2% early apoptotic cells and 50.1% late apoptotic cells (Figure 6A).

T47D cells treated with IDOE at 1.3 μg/mL induced apoptosis, with 0.5% early apoptotic cells and 32.7% late apoptotic cells, while control T47D cells showed 0.3% early apoptotic cells and 8% late apoptotic cells (Figure 6B).

### Increased caspase-3 expression levels in IDOE-treated tumor cells

**Figure 7:**
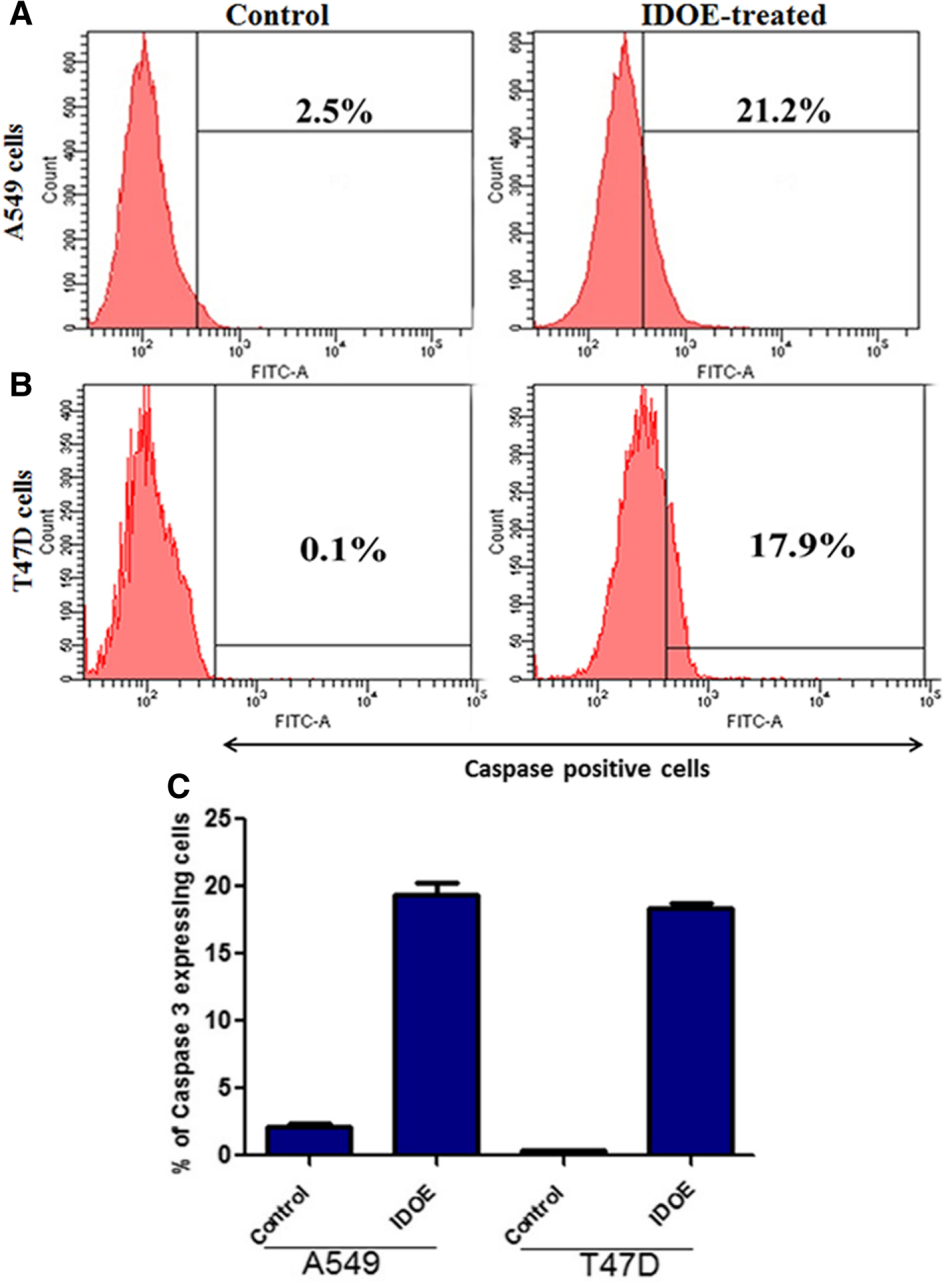
**Effect of IDOE on Caspase 3 expression. (A)** A549 cells, and **(B)** T47D cells. The cells were treated with IDOE and Caspase 3 was determined by flow cytometry. **(C)** Data expressed as mean ± SD from three independent experiments. *P* < 0.05 *vs* control.

Figure 7 shows the effects of IDOE at the level of caspase-3 induction in tumor cells. There was a significant increase in caspase-3 activity in the IDOE-treated cells compared with control cells after 48 h of treatment in both cell lines (*P* < 0.001).

### IDOE induces G2/M phase cell cycle arrest

**Figure 8:**
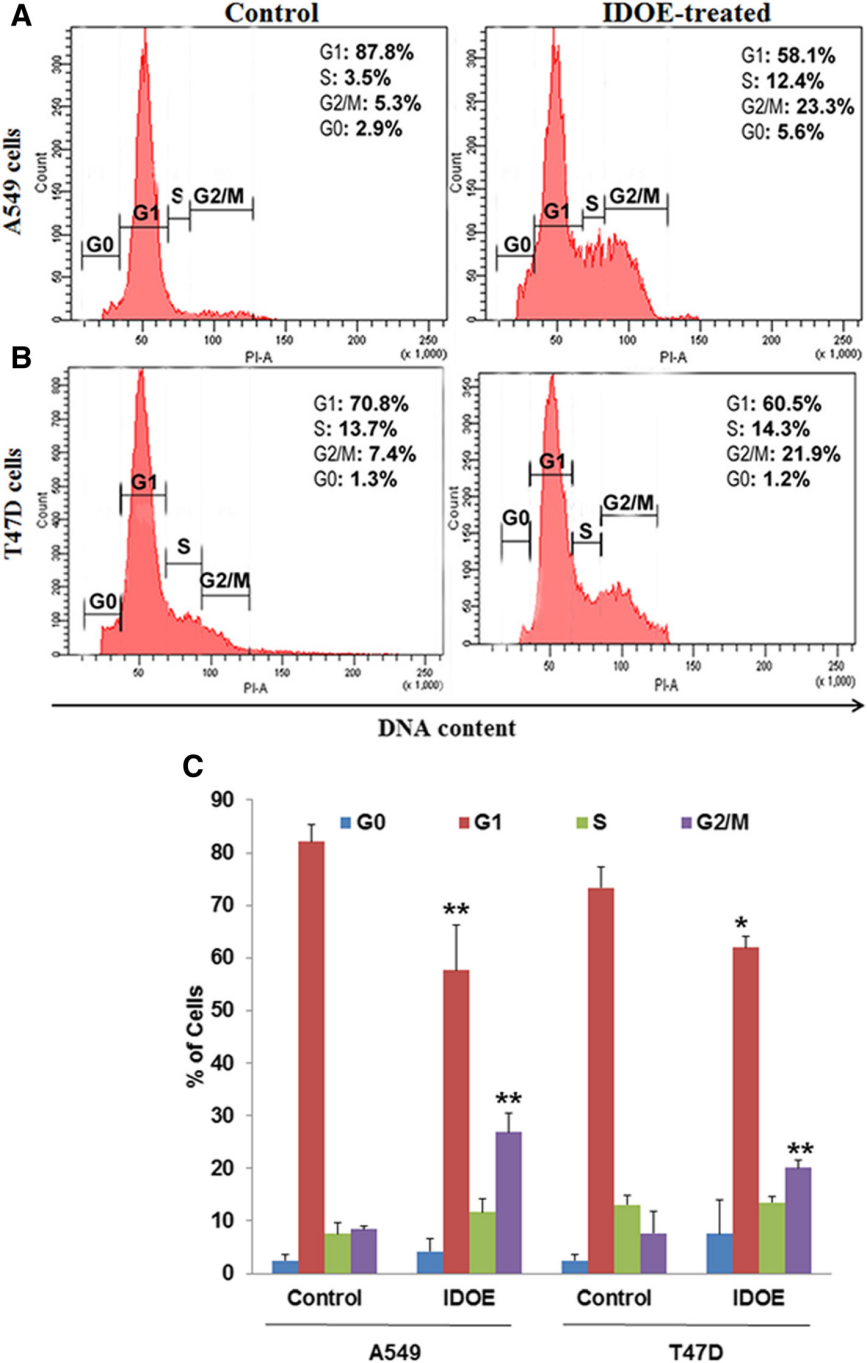
**Effect of IDOE on cell cycle progression. (A)** A549 cells **(B)** T47D cells. The cells were treated with IDOE for 48 h. The cell cycle distribution was determined by a flow cytometric analysis of the DNA content after staining with propidium iodide. **(C)** Data expressed as mean ± SD from three independent experiments. *P* < 0.05 *vs* control.

In A549 cells, the population of cells in G2/M phase increased from 5.3% to 23.3% following IDOE treatment for 48 h. Sub-G1 cells comprised 2.9% of the control cells, whereas treatment with IDOE at 10.46 μg/mL increased the cells in sub-G1 phase to 5.6% (Figure 8A). At a concentration of 1.3 μg/mL, IDOE significantly increased the population of T47D cells in G2/M phase (21.9%) (*P* = 0.0087) (Figure 8B). IDOE prevented G2/M transition and consequently induced G2/M phase arrest.

## Discussion

The basic structural requirement for the cytotoxic activity of a sesquiterpene lactone is the presence of an α-methylene-γ-lactone moiety in the molecule, regardless of the differences among structural types [12]. The presence of an α-methylene-γ-lactone moiety in IDOE may play a role in its anticancer effect. The molecular mechanism of IDOE underlying the antitumor activity remains unclear. This study aims to investigate the antiproliferative activity of IDOE toward breast carcinoma T47D cells and lung carcinoma A549 cells and its mechanism of action.

IDOE was non-cytotoxic to peripheral blood lymphocytes. These findings supported the previous report by Geetha et al. [11], indicating that IDOE showed specific cytotoxic activity toward cancer cells and not toward normal lymphocytes.

Many anticancer drugs function primarily to induce apoptosis in cancer cells and prevent tumor development [13,14]. The morphological changes of apoptosis observed in most cell types initially start with a reduction in cell volume and condensation of the nucleus [15]. Upon treatment with IDOE for 48 h, both A549 and T47D cells became detached from the monolayer and exhibited by nuclear fragmentation and chromatin condensation. The flow cytometry data obtained with annexin V/PI dual-staining confirmed that IDOE caused apoptosis in these tumor cells. To our knowledge, this is the first report on the apoptosis-inducing activity of IDOE for A549 and T47D tumor cells.

Activated caspases is triggered by signals from either death factors or mitochondrial alterations in the apoptotic process [16]. Activation of caspase-3 is the point of convergence of the intrinsic and extrinsic apoptotic pathways, leading to the common executive phase of apoptosis, and ultimately cell death. In our study, caspase-3 expression was significantly increased in IDOE-treated tumor cells compared with control cells.

Extensive DNA damage leads to activation of cell cycle check points and results in cell cycle arrest and apoptosis [17]. Accumulation of cells in the G2/M cell cycle phase was observed after 48 h of treatment with IDOE, indicating that the cells underwent G2/M arrest with this treatment in both cell lines.

## Conclusions

IDOE inhibited the proliferation of breast cancer cells and lung carcinoma cells and induced caspase-3-mediated apoptosis and cell cycle arrest in the treated cells.

## Abbreviations

IDOE: Isodeoxyelephantopin; DMSO: Dimethyl sulfoxide; PI: Propidium iodide; FBS: Fetal bovine serum; MTT: 3-(4,5-dimethylthiazol-2-yl)-2,5-diphenyltetrazolium bromide; PBS: Phosphate-buffered saline.

## Competing interest

All authors declare that they have no competing interest.

## Authors’ contributions

FAK and RP conceived and designed the study. GBS, MSN, LPG, FAK and DSR performed the experiments. FAK wrote the manuscript. All authors read and approved the final manuscript.
